# Therapeutic Potential of Marine-Derived Cyclic Peptides as Antiparasitic Agents

**DOI:** 10.3390/md21120609

**Published:** 2023-11-25

**Authors:** Ricardo Ribeiro, Lia Costa, Eugénia Pinto, Emília Sousa, Carla Fernandes

**Affiliations:** 1 Laboratório de Química Orgânica e Farmacêutica, Departamento de Ciências Químicas, Faculdade de Farmácia, Universidade do Porto, Rua de Jorge Viterbo Ferreira, 228, 4050-313 Porto, Portugal; ricardorc@live.com.pt (R.R.); liapdacosta@gmail.com (L.C.); esousa@ff.up.pt (E.S.); 2 Interdisciplinary Centre of Marine and Environmental Research (CIIMAR), Edifício do Terminal de Cruzeiros do Porto de Leixões, Av. General Norton de Matos s/n, 4050-208 Matosinhos, Portugal; epinto@ff.up.pt; 3 Laboratório de Microbiologia, Departamento de Ciências Biológicas, Faculdade de Farmácia, Universidade do Porto, Rua de Jorge Viterbo Ferreira 228, 4050-313 Porto, Portugal

**Keywords:** antiparasitic activity, cyclic peptides, leishmaniasis, malaria, marine resources, trypanosomiasis

## Abstract

Parasitic diseases still compromise human health. Some of the currently available therapeutic drugs have limitations considering their adverse effects, questionable efficacy, and long treatment, which have encouraged drug resistance. There is an urgent need to find new, safe, effective, and affordable antiparasitic drugs. Marine-derived cyclic peptides have been increasingly screened as candidates for developing new drugs. Therefore, in this review, a systematic analysis of the scientific literature was performed and 25 marine-derived cyclic peptides with antiparasitic activity (**1**–**25**) were found. Antimalarial activity is the most reported (51%), followed by antileishmanial (27%) and antitrypanosomal (20%) activities. Some compounds showed promising antiparasitic activity at the nM scale, being active against various parasites. The mechanisms of action and targets for some of the compounds have been investigated, revealing different strategies against parasites.

## 1. Introduction

Parasitic diseases affect millions of people around the world, especially in developing countries where sanitary and hygiene conditions are very poor, resulting in a huge mortality rate [1,2]. Despite this, the therapeutic arsenal available to treat this type of disease has been the same for many years, having low effectiveness and many side effects [3]. In addition to all these problems, the resistances acquired by parasites over time are also a huge concern [4]. The therapeutic scenario still remains the same because there is low investment in the development of this class of drugs since these diseases have a higher incidence in poor countries, which are not seen as good markets for the pharmaceutical industry [5]. Fortunately, this reality is changing thanks to the investment of some individuals and organizations for the development of antiparasitic drugs and to the cooperation between industry and academic research groups [6].

The parasitic diseases can be subdivided into two categories: those caused by protozoan parasites, such as malaria (caused by *Plasmodium*), leishmaniasis (caused by *Leishmania*), sleeping sickness, Chagas disease (caused by *Trypanosoma*), and toxoplasmosis (caused by *Toxoplasma gondii*); and those caused by helminths, such as schistosomiasis (caused by *Schistosoma mansoni*) and taeniiasis (caused by *Taenia solium*) [7].

Malaria is a severe infectious disease which is endemic in tropical and subtropical regions [8,9]. It can be caused by four species of the intracellular protozoan parasite from the *Plasmodium* family, with *P. falciparum* being the disease’s most dangerous form, and it is transmitted by mosquito bites [10]. According to the World Malaria Report 2022, in 2021, there were 247 million cases and 619,000 deaths from malaria. Moreover, malaria cases have been on the rise since 2016 [11].

Antimalarial drug options are still very limited [12]. One of the oldest drugs is quinine, whose discovery is considered serendipitous. Quinine was isolated from a cinchona tree (*Cinchona succirubra*), in 1820, by two French chemists. This compound began to be used as an antimalarial and its mechanism of action is still unknown. Quinine has a low therapeutic index and causes many side effects [13]. Subsequently, quinine analogues such as chloroquine, amodiaquine, primaquine, and mefloquine have emerged and are still used in therapy today; we highlight chloroquine, a 4-aminoquinoline derivative of quinine, which was synthesized in 1934 [12]. In 1970, artemisinin was isolated from the plant *Artemisia annua* and was a very useful compound for the treatment of malaria. In addition, to improve its pharmacokinetic and pharmacodynamic properties, molecular modifications were performed in artemisinin which gave rise to derivatives such as artesunate, arteether, and artemether [14]. All these compounds are still used to treat severe cases of malaria [12]. It is important to note that quinine and artemisinin derivatives are the main drugs currently used to treat malaria [15]. Derivatives of tetracyclines are sometimes used, such as doxycycline, which is used for treatment and prophylaxis in combination with quinine or artesunate, when the treatment with artesunate is not effective [15]. There is also the option of antifolate drugs, which are dihydrofolate reductase inhibitors; however, they are susceptible to rapid development of resistance by the parasite. Therefore, these drugs are used in combination to overcome the resistance issue [12]. The resistance to the few antimalarials available is caused by spontaneous mutations that increase the parasite’s tolerance to the drug [16]. Thus, although there is an urgent need to devise new strategies for developing new drugs that act on already known targets, it is also important to discover new targets. 

Currently, several strategies for developing new antimalarials are being explored [17]. For example, via synthesis, Wani et al. [18] obtained a methalocenic analogue of chloroquine, ferroquine, which is in clinical trials for the treatment of uncomplicated malaria [19]. Nature is also an immensely rich source of bioactive compounds, including antimalarials [20,21]. Natural products with antimalarial activity include various classes of compounds, such as alkaloids [22], terpenes [23], biflavonoids [24], lactones [25], coumarins [26], xanthones [27], quinones [28], and peptides [29,30]. Other strategies include the identification of new targets and the design of selective inhibitors for these targets [17] or the repositioning of drugs [31,32].

Leishmaniasis, a disease caused by 20 species of the protozoan parasite *Leishmania*, is transmitted through the bite of infected female sandflies. The intracellular parasite exists in two morphological forms, non-infectious promastigote and infectious amastigote [33]. This disease has three distinct forms: visceral, cutaneous, and mucocutaneous. Visceral leishmaniasis is the most serious form of the disease and is usually fatal if untreated; cutaneous leishmaniasis is the most common form of the disease and manifests itself through lesions and ulcers on the skin; and mucocutaneous leishmaniasis causes metastasis to the mucosal tissues of the nose and mouth [34]. According to the latest World Health Organization (WHO) report, there are an estimated 1 million new cases of leishmaniasis every year [35]. Leishmaniasis is considered one of the neglected tropical diseases that mostly affects developing countries [36,37].

The drugs currently used to treat leishmaniasis include pentavalent antimonials, amphotericin B, miltefosine, paromomycin, and pentamidine. The latter four are cases of drug repositioning [38]. All of these drugs, without exception, have many side effects and are associated with high toxicity, require long treatments, and are very expensive, and situations of resistance have already been reported [39]. Thus, there is an urgent need to develop new anti-leishmanial drugs with a better safety profile than current drugs, and that are effective against resistant species and affordable [40]. In recent years, several studies have been carried out in this area and different chemical families of compounds for antileishmanial activity have been explored, including benzimidazole derivatives [41], quinalidine derivatives [42], alkylphospholipid analogues [43], coumarins [44], chalcones [45], thienopyridine [46], and peptides [47,48,49].

Trypanosomiasis, a disease caused by trypanosomatids, is on the list of the six most dangerous tropical diseases according to WHO [2,50]. Depending on the parasite responsible for the disease, it is designed by different names: human African trypanosomiasis, also known as sleeping sickness, caused by two subspecies of *Trypanosoma brucei* (*T. b. gambiense* and *T. b. rhodesiense*), and American trypanosomiasis, also known as Chagas disease, caused by *Trypanosoma cruzi* [51,52]. 

Human African trypanosomiasis is transmitted to humans by the tsetse fly and is an endemic disease in sub-Saharan. This is a disease that affects people in rural areas and, if left untreated, can be fatal [53]. The drugs eflornithine, melarsoprol, nifurtimox, pentamidine, and suramin are used to treat this disease [54]. Nevertheless, these drugs have several limitations: they are expensive, have high toxicity, low oral bioavailability, and low efficacy, and require very long treatments [55]. Recently, fexinidazole has been approved for the treatment of trypanosomiasis caused by *T. b. gambiense* and is currently in clinical trials for trypanosomiasis caused by *T. b. rhodesiense* [56]. It is the first drug administered orally for the treatment of human African trypanosomiasis [57]. Moreover, acoziborole, also known as SCYX-7158, showed activity against *T. b. gambiense* and *T. b. rhodesiense* after a single oral administration [58,59]. In clinical trials, it proved to have good efficacy and a good safety profile [60]. Iheyamides A-C, naturally occurring peptides, exhibited antitrypanosomal activities against both *T. b. rhodesiense* and *T. b. brucei* (non-human pathogenic subspecies) [61].

American trypanosomiasis is a chronic systemic parasitic infection that is endemic in Latin America and affects poor rural areas [62,63]. This is a disease transmitted through the bite of the triatomine bug. By 2021, it was estimated that around 7 million people were infected and that around 65 million were at risk of infection [50]. The drugs benznidazole and nifurtimox are used to treat this disease [64]. Both drugs have several disadvantages, including adverse effects and their use being limited only to the acute phase of the disease, and resistance to these drugs has already been identified [65]. Trypanocidal drugs with greater efficacy and safety are therefore needed. Some research is underway in this direction. For example, some triazole derivatives used for fungal infections treatment have demonstrated in vitro and in vivo activity against *T. cruzi*, namely itraconazole [66], voriconazole [67], and ravuconazole [68]. The compound AN15368, which belongs to the oxaborole class of compounds, showed in vitro and in vivo activity against *T. cruzi* with low toxicity [69]. 

Toxoplasmosis, a disease caused by the parasite *Toxoplasma gondii*., is transmitted by eating meat containing *T. gondii* cysts or drinking water containing oocysts. This disease is found in Asia, Africa, America, and even Europe [70]. Currently, the therapeutic options for toxoplasmosis include the use of antiparasitic and antibacterial drugs. However, these drugs are only effective in acute toxoplasmosis and their use is dependent on factors such as intolerance, resistance, and side effects [71]. There are some compounds being studied that display activity against *T. gondii*, such as artemisinin derivatives [72], dihydrotriazines [73], fluoroquinolones derivatives [74], *N*-benzoyl-2-hydroxybenzamides [75], 4-(1*H*)-quinolones [76], and thiosemicarbazones [77].

The urgent need to develop new antiparasitic drugs is evident for a large number of reasons. Fortunately, academia and even some industries are already aware of the problem that infectious diseases represents and, in this sense, there are already some efforts and advances aimed at finding new effective, safe, cheap, and easy-to-administer drugs to combat what is a major global health problem that mainly affects developing countries. Among the various classes of naturally derived compounds that are being explored as new antiparasitic agents, the marine-derived cyclic peptides are included.

## 2. Marine-Derived Cyclic Peptides in Drug Discovery 

Marine organisms produce a wealth of natural products, creating a universe of unique and novel compounds [78]. Long-term evolution of marine organisms exposed to extreme conditions, such as pressure, temperature, light, and salinity, promote competitive advantages to their producers in their natural environments. Thus, many of the marine products have notable biological activities, including antiparasitic activity [79,80], making them good candidates for drug development [81]. Notable efforts have been carried out to discover new therapeutic entities based on marine natural products or their derivatives and, from 2015 to 2018, 17 clinically available drugs were reported, with 28 drugs in Phase I–III clinical trials [82]. Since then, 68 patents from marine organisms have been filed [83].

Naturally occurring and chemically modified marine peptides are of crucial relevance due to their unique structures, chemical properties, low inherent toxicity, and potential in a myriad of therapeutic areas [84,85], and they continue to fuel the drug pipeline [86]. Proof of this are the diverse examples of approved marine-derived peptides used in therapeutics. Ziconotide (Prialt^®^, analgesic drug) was the first Food and Drug Administration (FDA)-approved marine-derived peptide, in 2004 [87,88]. Other examples include brentuximab vedotin (Adcetris^®^), plitidepsin (Aplidin^®^), polatuzumab vedotin (Polivy^®^), tisotumab vedotin (TIVDAK^TM^), and disitamab vedotin (Aidixi^®^), all anticancer drugs [89]. We highlight that ziconotide and plitidepsin are both cyclic peptides. Marine-derived peptides attract great attention not only for the pharmaceutical field [90,91], but also for the cosmeceutical [92,93] and nutraceutical [94,95] industries.

Diverse marine organisms can produce peptides, such as bacteria [96], cyanobacteria [97], fungi [89,98], sponges [99], algae [100], and tunicates [101]. Some of them are found from symbionts [102] and non-symbiotic microorganisms [103,104]. One type of cyclic peptide often found in marine organisms includes the depsipeptides, molecules where one or more amide groups are replaced by the corresponding ester [105]. Cyclic depsipeptides have also contributed to expand the peptide chemical space [106].

Among the marine-derived peptides, cyclic peptides can offer some advantages compared to linear peptides, which make them attractive molecules to be explored. Most often, they present an increase in binding affinity and selectivity to the protein target [107]. This occurs because the cyclization reduces the spatial vibrations of the peptide molecule, lowering conformational changes, and increases the surface area available for interaction with the biotarget [108]. In addition to molecular recognition considerations, cyclization can also improve the absorption and membrane permeability of peptides because it reduces the energy barrier required for the peptide to adapt to the membrane environment and bind to transport proteins to enter the cell, both by passive diffusion or active transport [109]. Cyclic peptides also present greater metabolic stability, being resistant to the action of exopeptidases, due to the lack of terminal amine and carboxylic acid groups, and that of endopeptidases, by blocking the access to the cleavage site [110]. Structurally, cyclic peptides may comprise unique scaffolds, such as non-proteinogenic amino acids [111], or some structural modifications, including methylation [112], sulfuration [113], lipidation, and acetylation [114]. These characteristics have a key role in the interaction with biotargets [115,116].

Moreover, several synthetic derivatives of marine cyclic peptides and depsipeptides are also reported [117,118]. In fact, these naturally occurring compounds are very interesting models for molecular modifications to obtain more potent derivatives with improved properties and perform structure–activity relationship (SAR) studies [119,120]. 

Among the broad spectrum of bioactivities of natural and chemically modified marine peptides [121,122], anticancer [123] and antimicrobial [124] peptides are of pivotal relevance because they yield a very high output [125]. This review aims to gather the research findings on this class of marine compounds, particularly regarding their antiparasitic effects, through a comprehensive literature survey.

## 3. Marine-Derived Cyclic Peptides with Antiparasitic Effects

The research into marine-derived cyclic peptides with antiparasitic effects was designed with the aim of addressing the topic in a comprehensible approach. From this perspective, the research was systematized following the Preferred Reporting Items for Systematic Reviews and Meta-Analyses (PRISMA) protocol [126]. The identification of papers was conducted through a search of the SCOPUS database considering the following keywords or expressions: marine AND (peptide OR cyclic peptide OR cyclopeptide) AND (antiparasitic OR antimalarial OR antileishmanial OR antitrypanosomal). 

**Figure 1:**
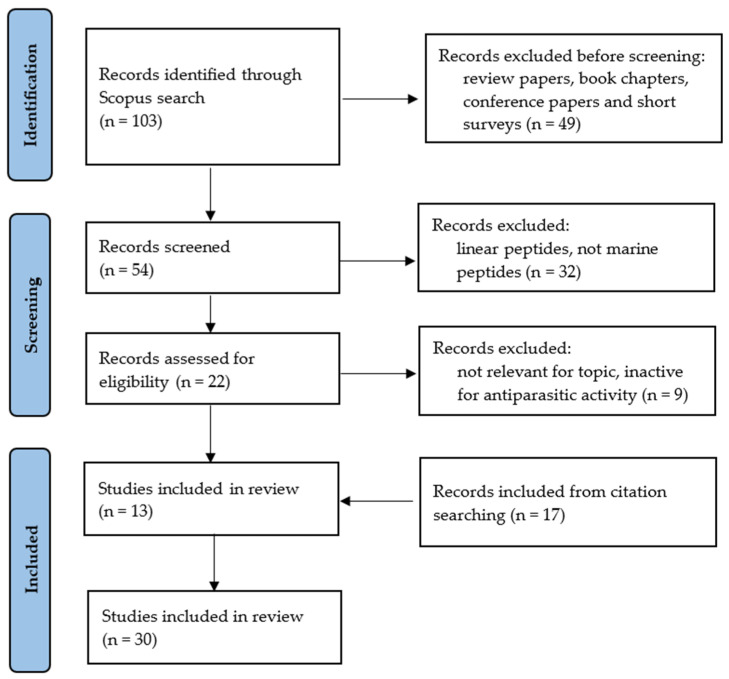
Flow diagram of literature search (n = number of scientific articles).

Inclusion criteria for selection of studies were all works published as original articles. The selection of studies took place in August/September 2023. Exclusion criteria considered the type of publication being removed: review papers, book chapters, conference papers, and short surveys. All the elected studies were published in English and addressed the theme of this research, and excluded the works describing linear peptides, peptides from sources other than marine, or inactive marine-derived cyclic peptides for antiparasitic activity, as well as studies not relevant to the topic. At the end, studies identified via other methods were also included, specifically from citation searching. All data collected were interpreted in a critical and impartial manner and the findings followed the recommendations of PRISMA. The methodological path that led to the selection of 30 scientific articles was outlined according to the flowchart shown in Figure 1. 

**Figure 2:**
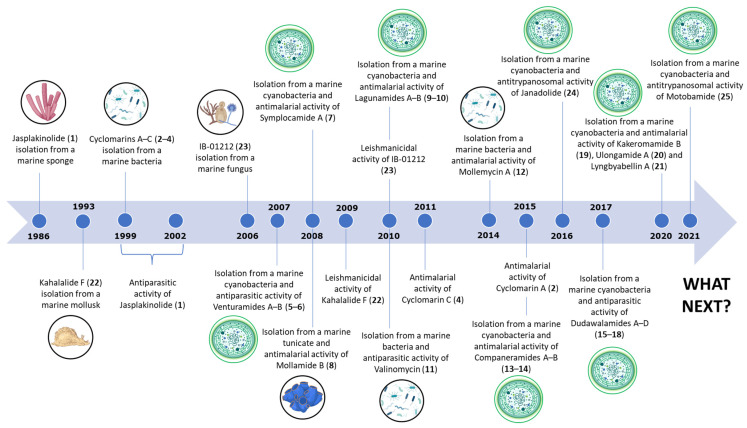
Timeline of antiparasitic marine-derived cyclic peptides research.

Through the comprehensive literature survey, 25 marine-derived cyclic peptides with antiparasitic activity (**1**–**25**) were found. In Figure 2, some of the most important landmarks of the history of marine-derived cyclic peptides with antiparasitic activity are summarized. Several marine sources were described, including cyanobacteria, bacteria, sponges, tunicates, fungi, and mollusks, with the marine-derived cyclic peptides from cyanobacteria being the most abundant. The main described antiparasitic activities associated with these peptides were antimalarial, antileishmanial, and antitrypanosomal. As shown in Figure 2, the decade between 2007 and 2017 was a period with significant research in this area. 

Table 1 summarizes the marine natural product name, cyclic peptides classification, compound producers, and antiparasitic activities for each compound and their references. 

In this section, marine-derived cyclic peptides are organized according to the main antiparasitic activity and, in each subsection, in chronological order of their discovery. Among them, 17 were found to be depsipeptides.

### 3.1. Malaria

**Figure 3:**
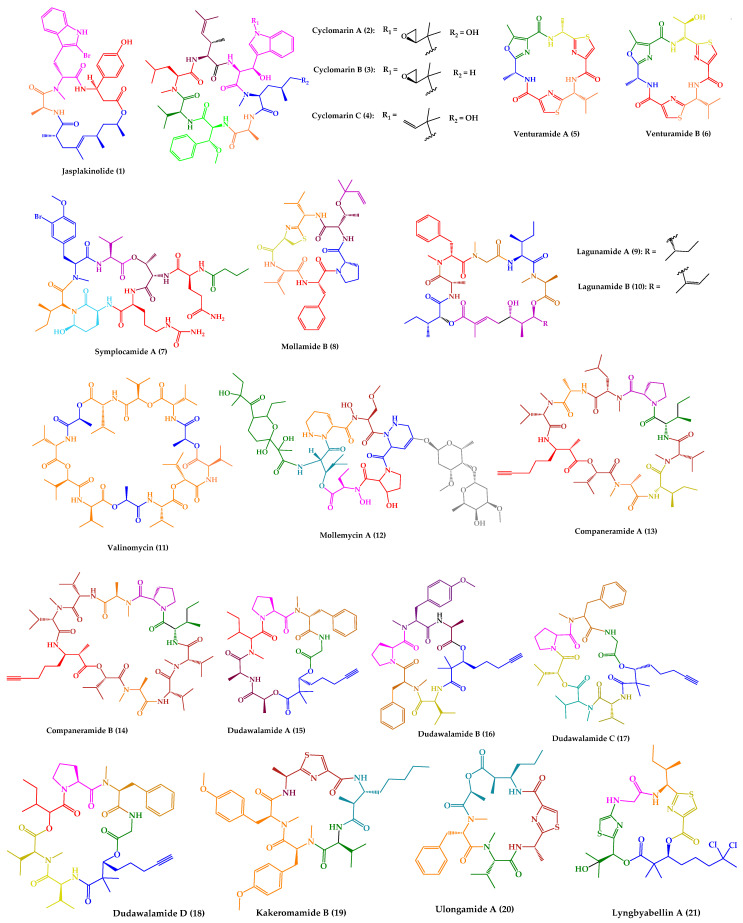
Structures of cyclic peptides with antimalarial activity (**1**–**21**).

One of the most described antiparasitic activities associated with marine-derived cyclic peptides is antimalarial activity [153]. Among the 25 marine-derived cyclic peptides, 21 peptides (**1**–**21**) exhibited promising results against *P. falciparum* (Figure 3). Some of them have also demonstrated activity against other parasites, or even displayed other biological activities.

Jasplakinolide (**1**), also named jaspamide, was isolated from the soft-bodied sponge species Jaspis collected off the shore of the island of Benga, Fiji [127]. This 19-membered macrocyclic depsipeptide, containing a macrolactam joined by a tripeptide unit and a non-peptide polypropionate sector (Figure 3), has been widely studied over the years. Diverse synthetic strategies for its total synthesis and analogs were explored, which were summarized in a recent review [154]. 

Regarding the biological activities, several studies can be found related to various activities of **1**, such as antifungal [155,156], antitumor [157,158,159,160,161], neuroprotective [162], and antiparasitic [128,129,130] activities. Jasplakinolide (**1**) is a potent inducer of actin polymerization and filament-stabilizing drug [163,164], and this capability is relevant for its biological effects [165,166,167,168,169,170]. Compound **1** dramatically reduces the critical concentration of actin subunits necessary to drive polymerization and stabilizes filaments [163,170].

Regarding antiparasitic activity, it was found that **1** inhibits *P. falciparum* growth and impairs host cell invasion due to the stabilization of parasite actin filaments, in a time- and concentration-dependent manner. The decrease was remarkable at day 2 at concentrations of 0.3 µM and above, and parasites finally disappeared at day 4 [128]. *P. falciparum* and other parasites actively invade host cells, using a mechanism that depends on the interaction of the motor protein myosin and actin filaments which serve as tracks [171,172]. Thus, it has been proposed that the unstable nature of apicomplexan actin filaments is essential for parasite survival [171].

**Figure 4:**
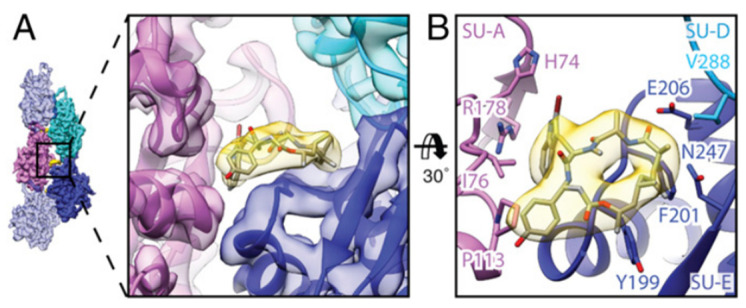
Interaction of jasplakinolide (**1**) with PfAct1. (**A**) **1** (yellow) binds noncovalently to three actin subunits (magenta, blue, and cyan). (**B**) Tilted top view of **1** and amino acids involved in the interaction [173].

By electron cryomicroscopy, the near-atomic structure of jasplakinolide (**1**)-stabilized *P. falciparum* actin 1 filaments (PfAct1) was determined. Jasplakinolide (**1**) binds at regular intervals inside the filament to three adjacent actin subunits, reinforcing filament stability by hydrophobic interactions (Figure 4) [173]. 

In a previous study, jasplakinolide (**1**) also drastically decreased the *T. gondii* motility and host cell invasiveness [129]. Additionally, **1** induced an acrosomal process and allowed better visualization of the actin filaments, revealing the conoid and apical complex as major sites of actin polymerization [174]. 

Jasplakinolide (**1**) has also been reported to inhibit both the growth and encystation of *Entamoeba histolytica* and *Entamoeba invadens*, inducing the formation of F-actin aggregates [130,131]. Compound **1** inhibited the growth of *E. histolytica* in a dose-dependent manner, in which 0.1 and 0.3 mM of the drug had a similar inhibitory effect, whereas 0.5, 0.7, and 1 mM inhibited 62, 82, and 100% of control growth, respectively. The inhibition of *E. invadens* by 1 mM of **1** was 25% [131].

Cyclomarins A–C (**2**–**4**) were isolated from extracts of a cultured marine bacterium *Streptomyces* sp. CNB-982, collected in the vicinity of San Diego, CA. Cyclomarin A (**2**) is the major metabolite, while cyclomarins B and C (**3**–**4**) are only produced in lower percentages (2–3%). Cyclomarins A–C (**2**–**4**) are cyclic heptapeptides containing two proteinogenic amino acids, (*S*)-Ala and (*S*)-Val), an *N*-methylated (*S*)-Leu, in addition to four unusual (*S*)-amino acids (Figure 3). The planar structure of **2**–**4** was elucidated by 1D and 2D nuclear magnetic resonance (NMR) methods, and their stereochemistry was determined by X-ray crystallography of a diacetate derivative of cyclomarin A (**2**) [132]. 

**Figure 5:**
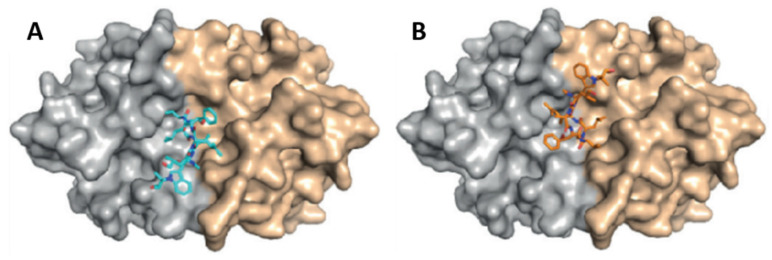
Crystal structure of PfAp3Aase in complex with cyclomarin A (**2**) (PDB ID: 5CS2). Surface representation of the PfAp3Aase homodimer with the two monomers colored in gold and grey with the fitted cyclomarin A molecules shown as a ball-and-stick model in cyan (**A**) and orange (**B**) for the two, mutually exclusive orientations [133].

Detailed studies at Novartis indicated a strong activity of compound **2** against *P. falciparum* strain NF54 in a nanomolar range, with a half-maximal inhibitory concentration (IC_50_) value of 40 nM [133]. By chemical proteomics, the biotarget was identified to be the diadenosine triphosphate hydrolase (PfAp3Aase) of the protozoan parasite, without affecting the human homolog hFHIT. Co-crystallization experiments revealed that one molecule of **2** binds a dimeric PfAp3Aase and prevents the formation of the enzyme–substrate complex (Figure 5) [133]. Cyclomarin C (**4**) has also been isolated from *Streptomyces* sp. BCC26924 and exhibited antimalarial activity against the multidrug-resistant *P. falciparum* K1 strain, with IC_50_ values of 0.24 µg/mL [134].

In addition to antiparasitic activity, cyclomarin A (**2**) also displayed significant anti-inflammatory activity in both in vivo and in vitro assays [132] and anti-tuberculosis activity (minimum inhibitory concentration (MIC) of 0.1 μM) by targeting the ClpC1 subunit of the caseinolytic protease [175,176]. 

Regarding the outstanding biological activities of cyclomarins, it is not surprising that synthetic studies have been performed to obtain these compounds in higher quantity as well as analogues for SAR studies [177,178]. The first report of stereoselective syntheses of four unusual amino acids constituents of cyclomarin A (**2**) was described by Yokokawa and co-workers [179], in 2002. A few years later, synthetic access to cyclomarin C (**4**) was achieved and optimized by Yao and co-workers [180,181]. Just over ten years later in 2016, Barbie and Kazmaier [182,183] accomplished the total synthesis of cyclomarin A (**1**) for the first time. They also reported the total synthesis of cyclomarin C (**4**), cyclomarin D, and other natural peptides [182]. Regarding the planning of derivatives, the first strategy was via semi-synthesis [176], and then by total synthesis [184]. Some addressed simplified structures, such as deoxycyclomarin C [185].

Venturamides A–B (**5**–**6**) were isolated from the crude organic extract of a Panamanian collection of *Oscillatoria* sp. from Buenaventura Bay, via an antimalarial bioassay-guided fractionation. Their isolation constitutes the first example of the identification of cyanobacterial peptides with selective antimalarial activity. Venturamides A–B (**5**–**6**) comprise 2,4-disubstituted thiazole units (Figure 3), and their complete structure elucidation was determined via 1D and 2D NMR analyses and Marfey’s method [135]. Venturamides A–B (**5**–**6**) were tested for their antimalarial activity against the W2 chloroquine-resistant strain of the malaria parasite. Compound **5** exhibited an IC_50_ value of 8.2 µM against *P. falciparum*, with only mild cytotoxicity to mammalian Vero cells (IC_50_ value of 86 µM). Compound **6** also displayed effective antimalarial activity against *P. falciparum*, with an IC_50_ value of 5.6 µM, and mild cytotoxicity to mammalian Vero cells, with an IC_50_ value of 56 µM. The positive control, chloroquine, showed IC_50_ values of 80–100 nM [135]. Liu et al. [186] described the total synthesis of both compounds via an effective one-pot procedure for enantiomerical synthesis of thiazole-containing amino acids.

Symplocamide A (**7**) (Figure 3) was isolated from the marine cyanobacterium *Symploca* sp., collected from Sunday Island in Papua New Guinea. The planar structure was elucidated by detailed NMR and MS analysis, and chiral high performance liquid chromatography (HPLC) was the method used for stereochemical elucidation of amino acid residues. Compound **7** was screened against three tropical parasites, specifically malaria, Chagas disease, and leishmaniasis, showing interesting results. An IC_50_ value of 0.95 µM was obtained regarding the inhibition of W2 *P. falciparum*, while for both *T. cruzi* and *L. donovani*, both IC_50_ values were higher than 9.5 µM [136]. Symplocamide A (**7**) is also a potent cancer cell cytotoxin to H-460 lung cancer cells, with an IC_50_ value of 40 nM, and neuro-2a neuroblastoma cells, with an IC_50_ value of 29 nM. Compound **7** also inhibits serine proteases with a 200-fold greater inhibition of chymotrypsin over trypsin [136]. The total synthesis of symplocamide A (**7**) using a solid-phase strategy was reported [187].

Mollamide B (**8**) (Figure 3) was isolated from the tunicate *Didemnum molle*, collected from Manado Bay, Indonesia. The planar structure was established using 1D and 2D NMR experiments. The absolute configurations of amino acid residues were assigned by Marfey’s method, while the relative configuration at the thiazoline moiety was determined using molecular modeling coupled with NMR-derived restraints. Mollamide B (**8**) showed inhibitory activity against *P. falciparum*, clones D6 and W2, with IC_50_ values of 2.0 and 21 µg/mL, respectively. Mollamide B (**8**) also exhibited slight activity against L. donovani, with an IC_50_ value of 18 µg/mL [137]. 

The cyclic pentadepsipeptides lagunamides A–B (**9**–**10**) (Figure 3) were isolated from the marine cyanobacteria *Lyngbya majuscula* obtained from Pulau Hantu Besar, Singapore. Extensive spectroscopic analysis, including 2D NMR experiments, in addition to Marfey’s method and ^3^J_H-H_ coupling constant values, a modified method based on Mosher’s reagents, and analysis using liquid chromatography–mass spectrometry (LC–MS), allowed the total elucidation of these molecules. Compounds **9**–**10** displayed in vitro antimalarial properties, with IC_50_ values of 0.19 µM and 0.91 µM, respectively, against *P. falciparum*. Curiously, the only structural difference between these compounds is an additional olefinic group between C_40_ and C_41_ in **10**. Consequently, this slight structural difference is responsible for an increase in antimalarial activity observed for **9** [138]. 

In addition to antimalarial activity, lagunamides A–B (**9**–**10**) displayed antiswarming activity when tested at 100 ppm against the Gram-negative bacterial strain *Pseudomonas aeruginosa*, which exerted 62% for **9** and 56% for **10**, compared to control. Compounds **9**–**10** also exhibited potent cytotoxic activity against P388 murine leukemia cell lines, with IC_50_ values of 6.4 and 20.5 nM, respectively [138]. Further studies revealed that **9** exhibited a selective growth inhibitory activity against a panel of cancer cell lines, with IC_50_ values ranging from 1.6 nM to 6.4 nM [188]. Molecular mechanism studies suggested that the cytotoxic effect of these compounds might be via induction of mitochondrial mediated apoptosis [188,189].

The total synthesis and stereochemical revision of lagunamide A (**9**) was first described by Dai et al. [190]. Later, Lin and co-workers [191] reported the synthesis of **9** and five analogues. Although the total synthesis of lagunamide B (**10**) has not yet been described, a synthetic approach toward the total synthesis of a lagunamide B (**10**) analogue was reported [192].

A cyclic dodecadepsipeptide, valinomycin (**11**) (Figure 3), was identified from *Streptomyces* sp. strains isolated from Mediterranean sponges collected by SCUBA diving offshore of Rovinj, Croatia [140]. This was the first report of the isolation of **11** from a marine source [140]. Nevertheless, **11** had already been recovered from various soil-derived actinomycetes, being first reported in 1955 [193]. Over the years, this cyclic depsipeptide has been extensively studied and a wide range of issues were explored, including structural characterization, biogenesis, synthesis, and bioactivity. In a Scopus search (https://www.scopus.com, accessed on 22 October 2023, with the keyword “valinomycin” and searching within “article title”, 717 original articles were found. Recently, a review summarizing all the relevant features concerning this cyclic depsipeptide (**11**) was published [194]. Considering the subject of this review, it is important to highlight that **11** showed antiparasitic activity against *P. falciparum*, with an IC_50_ value of 5.3 ng/mL [139]. Moreover, **11** also exhibited inhibitory activity against both *L. major*, with an IC_50_ value lower than 0.11 μM, and *T. brucei*, with an IC_50_ of 3.2 nM [140]. Other relevant biological activities have been described for valinomycin [194], such as insecticidal [195], antiviral [196,197], antibacterial [197], antifungal [198], and antitumor [199] activities. Recent studies reported that **11**, as a mitophagy activator, also played a positive role in the treatment of Parkinson’s and Alzheimer’s diseases [200,201].

A glyco-hexadepsipeptide-polyketide, mollemycin A (**12**) (Figure 3), was isolated from a marine-derived *Streptomyces* sp. (CMB-M0244) collected off South Molle Island, Queensland. The structure of **12** was elucidated by detailed spectroscopic analysis, supported by chemical derivatization and degradation. C3 Marfey’s analysis was performed for stereochemical configuration assignments. Mollemycin A (**12**) exhibited exceptionally potent and selective growth inhibitory activity against drug-sensitive 3D7 and multidrug-resistant Dd2 clones of *P. falciparum*, with IC_50_ values of 7 and 9 nM, respectively. Remarkably, compound **12** exhibited greater activity when compared to the positive control, chloroquine, with IC_50_ values of 13 nM for 3D7 and 130 nM for Dd2. Moreover, lower cytotoxicity against a mammalian cell line (>20-fold) was observed [141].

Mollemycin A (**12**) also exhibited exceptionally potent and selective growth inhibitory activity against Gram-positive and Gram-negative bacteria (IC_50_ = 10–50 nM) [141].

Companeramides A–B (**13**–**14**) were isolated from a marine cyanobacterial assemblage comprising a small filament *Leptolyngbya* sp., from a reef pinnacle in Coiba National Park, Panama. Companeramides A–B (**13**–**14**) contain eight α-amino acid units, a 3-amino-2-methyl-7-octynoic acid, and hydroxy isovaleric acid (Figure 3). Their planar structures were elucidated by NMR spectroscopy and MS. The absolute configurations of the amino and hydroxy acid units in both compounds were determined using a combination of Marfey’s method and chiral HPLC. Companeramides A–B (**13**–**14**) were tested in vitro against three strains of the malaria parasite *P. falciparum*, D6, Dd2, and 7G8, showing high antiplasmodial activity, with IC_50_ values ranging from 0.22 to 0.70 µM for **13** and from 0.57 to 1.10 µM for **14**. The positive control, chloroquine, showed IC_50_ values of 5 to 80 nM [142].

Dudawalamides A–D (**15**–**18**) were isolated from a Papua New Guinean field collection of the cyanobacterium *Moorea producens*, by a combination of bioassay-guided and spectroscopic approaches. Experiments using 1D and 2D NMR and MS analysis were performed for planar structure elucidation. Diverse techniques were used for the absolute configuration assignments, namely X-ray crystallography, modified Marfey’s analysis, chiral gas chromatography (GC)-MS, and chiral HPLC. Structurally, they consist of six amino acids and a 2,2-dimethyl-3-hydroxy-7-octynoic acid moiety (Figure 3). Dudawalamides A (**15**) and D (**18**) showed the most potent activities against *P. falciparum*, with IC_50_ values of 3.6 and 3.5 μM, respectively. Dudawalamides B (**16**) and C (**17**) were significantly less potent than **15** and **18** [143]. All exhibited minimal mammalian cell cytotoxicity. Regarding SAR features, it was found that slight changes in configuration and sequence of residues had a significant effect on the bioactivity of these marine cyclic peptides. For example, dudawalamides C (**17**) and D (**18**) only differ in one methyl group at one residue, specifically L-Hiva to Dallo-Hmpa; however, the single methyl group and stereochemical inversion resulted in a more than 3-fold difference in their *P. falciparum* inhibition [143]. It was found that dudawalamide D (**18**) was also relatively potent against *L. donovani* (IC_50_ = 2.6 μM) [143]. 

Recently, a new cyclic peptide, kakeromamide B (**19**) (Figure 3), was isolated from an extract of a marine cyanobacterium *Moorea producens* collected off the Northern Lau Islands of Fiji. The extract showed strong potency against *P. falciparum* and low toxicity to human liver cells [144]. The stereostructure of kakeromamide B (**19**) was assigned by different spectroscopic techniques, high-resolution electrospray ionization mass spectrometry (HRESIMS), and Marfey’s analysis. Kakeromamide B (**19**) exhibited activity against *P. falciparum* blood-stage and against *P. berghei* liver schizonts with effective concentration in 50% of population (EC_50_) values of 0.89 and 1.1 µM, respectively. By a threading-based computational method, FINDSITEcomb2.0, the binding of **19** to potentially druggable proteins of *P. falciparum* was predicted. Kakeromamide B (**19**) was predicted to bind to several *Plasmodium* actin-like proteins and a sortilin protein, suggesting possible interference with parasite invasion of host cells. In a mammalian actin polymerization assay, it was found that **19**, in fact, stimulated actin polymerization in a dose-dependent manner [144].

The cyclic depsipeptides ulongamide A (**20**) and lyngbyabellin A (**21**) (Figure 3) were also identified from the same antimalarial extract of the Fijian marine cyanobacterium *Moorea producens* [144]. Ulongamide A (**20**) was previously isolated from Palauan collections of the marine cyanobacterium *Lyngbya* sp. [202] and lyngbyabellin A (**21**) from the marine cyanobacterium *Lyngbya majuscula* [203]. While **20** exhibited moderate activity against *P. falciparum* blood-stages with EC_50_ values of 0.99 µM, **21** was more potent with an EC_50_ value of 0.15 nM [144].

### 3.2. Leishmaniasis

**Figure 6:**
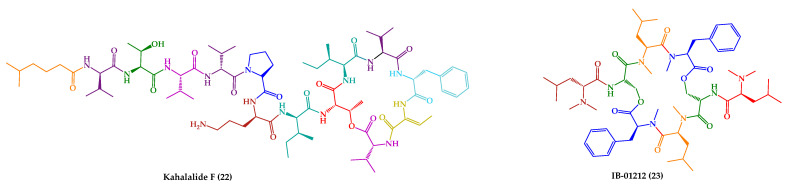
Structures of cyclic peptides with leishmanicidal activity (**22**–**23**).

Some marine-derived cyclic peptides with antileishmanial activity have already been presented in the previous subsection, as they also exhibited antimalarial activity, namely venturamides A–B (**5**–**6**), symplocamide A (**7**), mollamide B (**8**), valinomycin (**11**), and dudawalamides A–D (**15**–**18**). In this subsection, two natural products **22** and **23** (Figure 6) are described, which proved to be very promising for the treatment of leishmania and whose mechanism of action has been investigated. In addition, analogues were synthesized with the aim of obtaining more potent compounds and performing SAR studies.

Kahalalide F (**22**), one of the most widely studied marine-derived peptides [204], was isolated from green alga metabolites that are eaten by the sacoglossan mollusk, *Elysia rufescens* [146]. Kahalalide F (**22**) is the most promising compound of the kahalalide family, which includes cyclic depsipeptides with variable size and peptide series, ranging from C_31_ tripeptide to C_75_ tridecapeptide containing different fatty acid chains [145]. Kahalalide F (**22**) comprises 14 residues, 5 of which form a 19-membered ring (Figure 6), and its absolute stereochemistry was accessed after extensive hydrolytic trials, and a combination of acid hydrolysis and hydrazinolysis [205]. Nevertheless, the absolute configurations of some amino acid residues were not consensual [206,207]. Its synthesis has been described by a solid-phase synthetic approach and solution macrocyclization [206,208]. Several new improved synthetic routes were explored based on convergent approaches with distinct orthogonal protection schemes [209]. Considering the high cost in producing analogues via solid-phase synthesis, a degradation and reconstruction approach was explored using natural kahalalide F (**22**) from a seasonal algal bloom for the generation of semisynthetic libraries [210].

Kahalalide F (**22**) is notable mainly because its antitumor activity against a panel of human prostate and breast cancer cell lines [211], and several molecular mechanisms can be responsible for its rapid and potent cytotoxicity, including changes in lysosome morphology [212,213], inhibition of the ErbB3 signaling pathways [213,214,215], induction of oncosis [216], and alteration of the cell membrane permeability [217]. Kahalalide F (**22**) was one of the first generation of drugs from the sea to undergo clinical trials, along with Yondelis, Aplidin, ES285, and Zalypsis [211,218]. Kahalalide F (**22**) completed its safety evaluation in Phase I clinical trials in patients with various advanced solid tumors [219,220,221]. Unfortunately, in Phase II clinical trials, a lack of efficacy was found [222].

Considering its high potential as a cytotoxic drug candidate, several attempts have been made to modify the structure of kahalalide F (**22**) to produce analogues with a higher potency, longer half-life, or better delivery [223]. For example, new analogues were synthesized and conjugated in gold nanoparticles with enhanced in vitro antitumor activity [224].

**Figure 7:**
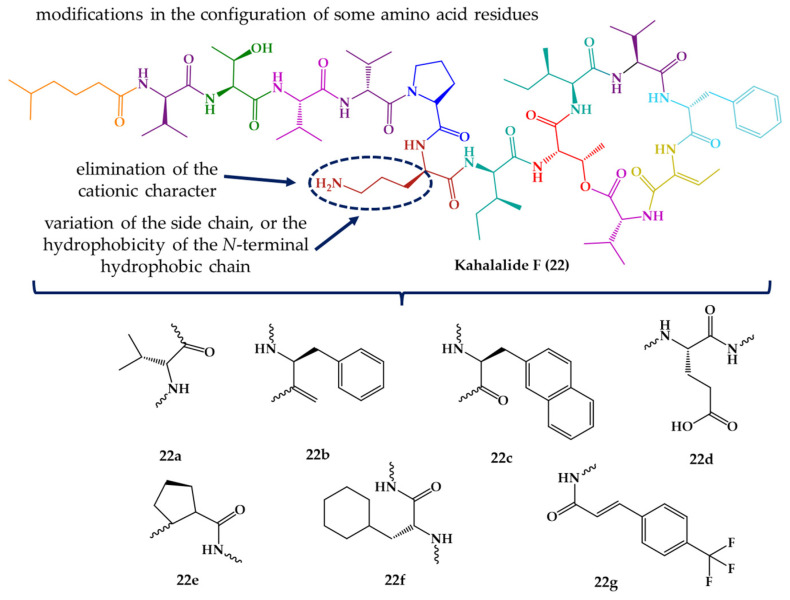
Rationale for modifications in kahalalide F (**22**) and structures of synthetic analogues (**22a**–**g**).

It is important to highlight that kahalalide F (**22**) and analogues were also tested for other activities, including leishmanicidal activity. A series of analogues were synthetized, preserving the core structure of kahalalide F (**22**), while changing specific amino acid residues to explore their influence on the biological activity (Figure 7) [147].

All compounds were assayed against promastigote and amastigote stages of *Leishmania* and, in general, amastigotes were more resistant than promastigotes to kahalalide F (**22**) and analogues. The concentrations at which the proliferation of the parasites was inhibited by 50% (LC_50_) were 6.13, 8.31, and 29.53 μM for kahalalide F (**22**), against *L. donovani* (promastigote), *L. pifanoi* (promastigote), and *L. pifanoi* (amastigote), respectively [147]. Analogue **22c** was the most interesting compound, with LC_50_ values of 3.04, 5.82 and 5.01 μM, against *L. donovani*, *L. pifanoi*, and *L. pifanoi*, respectively. The positive control, amphotericin B, showed LC_50_ values of 0.08–0.2 μM [147].

**Figure 8:**
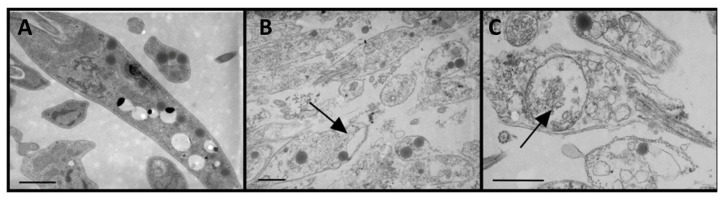
Electron microscopy of *L. donovani* promastigotes treated with analogue **22c** in a concentration that caused 95% lethality (5 µM). (**A**): control parasites; (**B**,**C**): promastigotes treated with **22c**. Arrows highlight the formation of large vacuoles inside the parasites (Reprint with permission from [147], Copyright (2009) American Chemical Society).

Electron microscopy of *L. donovani* promastigote parasites treated with **22c** evidenced severe morphological damage to the membrane of the parasite, indicating a full permeabilization of the parasite plasma membrane, with the cytoplasm full of vesicles translucent to electrons and large vacuoles (Figure 8). It was found that the permeability alteration of the plasma membrane of the parasite was strongly associated with the lethality of kahalalide F (**22**) and active analogues, which correlates significantly with their leishmanicidal activity [147].

Regarding SAR features, is important to note that a net cationic character and the maintenance of the configuration of selected residues was required for the leishmanicidal activity [147]. In addition, kahalalide F (**22**) and some analogues also exhibited antifungal activity against yeasts (*Candida albicans* and *Cryptococcus neoformans*) and filamentous fungi (*Aspergillus fumigatus* and *Fusarium* spp.), among others [213,225,226].

IB-01212 (**23**) (Figure 6), a cyclodepsipeptide isolated from the mycelium of the marine fungus *Clonostachys* sp. ESNA-A009 [148], also showed leishmanicidal activity at a low micromolar range of concentrations on promastigote and amastigote forms of the parasite [149]. The structure of IB-01212 (**23**) and the configurations of amino acid residues were assigned by spectroscopy techniques and a combination of Marfey and menthol methods [148]. 

The total synthesis of IB-01212 (**23**) [227] and analogues [228] was reported by the same research group. Recently, IB-01212 (**23**) was used as model to investigate a facile synthesis process of *N*-methylated peptides via simultaneous *N*-methylation of several peptide bonds in the presence of peptide bonds that were not to be methylated. The aim was to improve the potency and physicochemical properties, especially membrane permeability [229].

Before being studied for *Leishmania*, IB-01212 (**23**) and synthetic analogues proved to be highly cytotoxic to different tumor cell lines [148,228]. Regarding leishmanicidal activity, they were demonstrated to be effective on both parasite forms, with LC_50_ values of 5.9–25.9 μM and 10.5–49.1 μM for amastigotes and promastigotes, respectively. It was found that all were more active on the amastigotes, the pathological form in vertebrates of *Leishmania*, and IB-01212 (**23**) exhibited the highest activity on promastigotes [149]. SAR studies revealed that the most active analogue against the amastigote form of the parasite contained a 22-atom cycle, amide and ester bonds, and C-2 asymmetry. The mechanism of action for leishmanicidal activity was associated with the depolarization of the mitochondrial electrochemical gradient, which, in some cases, caused the death of the parasite through an apoptotic-like process [149].

### 3.3. Trypanosomiasis

**Figure 9:**
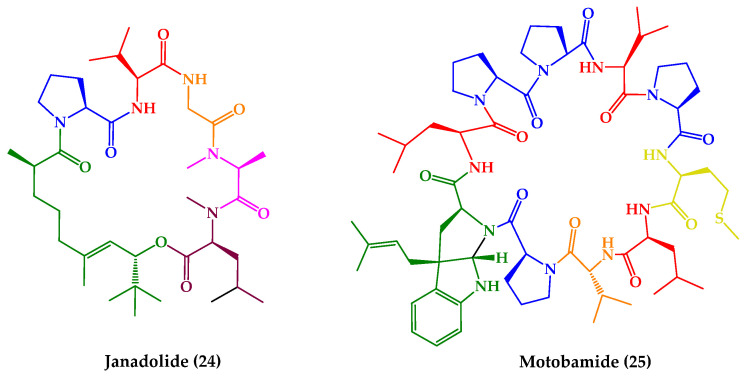
Structures of cyclic peptides with antitrypanosomal activity (**24**–**25**).

The previously described marine-derived cyclic peptides venturamides A–B (**5**–**6**), symplocamide A (**7**), valinomycin (**11**), and dudawalamide D (**18**), in addition to other antiparasitic effects, also exhibited antitrypanosomal activity. In this subsection, two additional compounds (**24**–**25**) (Figure 9) displaying antitrypanosomal activity are described, which demonstrated activity against the two subspecies of *T. brucei* (human African trypanosomiasis or sleeping sickness) and/or *T. cruzi* (American trypanosomiasis or Chagas disease).

**Figure 10:**
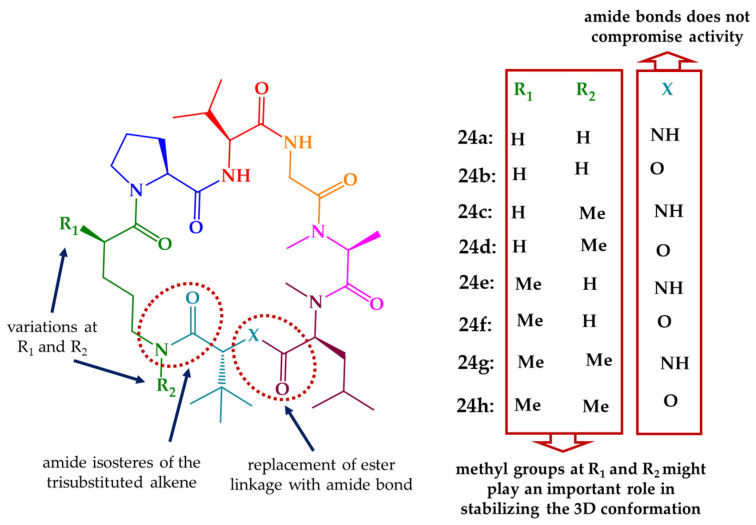
Rationale for modifications in janadolide (**24**) and structures of simplified analogues (**24a**–**h**).

Janadolide (**24**), a cyclic polyketide–peptide hybrid possessing a tert-butyl group (Figure 9), was isolated from an *Okeania* sp. marine cyanobacteria that was collected at the coast near Janado, Okinawa. The planar structure **24** was determined via spectroscopic analyses, and the absolute configurations of the amino acid residues via chiral LC. Regarding the polyketide moiety, the stereochemical assignment of the two stereogenic centers was performed based on a combination of degradation reactions and spectroscopic analyses. Compound **24** showed strong antitrypanosomal activity against *T. b. brucei* GUTat 3.1 strain with an IC_50_ value of 47 nM, without cytotoxicity against human cells (such as MRC-5, HL60, and HeLa cells) at 10 μM. Remarkably, it was found that **24** was more effective than suramin (IC_50_ value of 1.2 μM), a commonly used therapeutic drug [150]. Two years after its discovery, janadolide (**24**) was synthetized by Ojima et al. [230]. Moreover, a des-tert-butyl analogue of **24** was also reported but with reduced activity against *T. b. brucei* [231]. More recently, the total synthesis of **24** along with eight analogues (**24a**–**h**), bearing a simplified polyketide motif (Figure 10), was described via a solid-phase strategy for the linear peptide precursor and solution macrocyclization [151]. All compounds were tested against the pathogenic STIB 900 strain of *T. b. rhodesiense* and the Tulahuen C4 strain of *T. cruzi*, with IC_50_ values ranging from 33 to 104 μM. None of the compounds displayed cytotoxicity against human L6 cell lines up to a concentration of 100–150 μM [151].

Recently, motobamide (**25**) (Figure 9) was isolated from a marine cyanobacterium, *Leptolyngbya* sp., which was collected at Bise, Okinawa Island, Okinawa Prefecture, Japan. The planar structure of **25** was elucidated based on extensive analysis of 1D and 2D NMR and high-resolution (HR) MS spectra. The absolute configurations of all amino acids were assigned by chiral LC analysis, except for a prenyl-tryptophan residue, which was determined by correlations of NOESY and comparison of the calculated and experimental electronic circular dichroism (ECD) spectra. Motobamide (**25**) was found to inhibit the growth of bloodstream forms of *T. b. rhodesiense* strains IL-1501, with an IC_50_ value of 2.3 μM. Additionally, the cytotoxicity of **25** against normal human fibroblasts WI-38 cells was more than 20-fold weaker, with IC_50_ value of 55 μM [152].

### 3.4. Final Remarks

From the systematic analysis of the scientific literature, 25 marine-derived cyclic peptides with antiparasitic activity (**1**–**25**) were found. Several marine sources were described, with cyanobacteria being the main resource.

**Figure 11:**
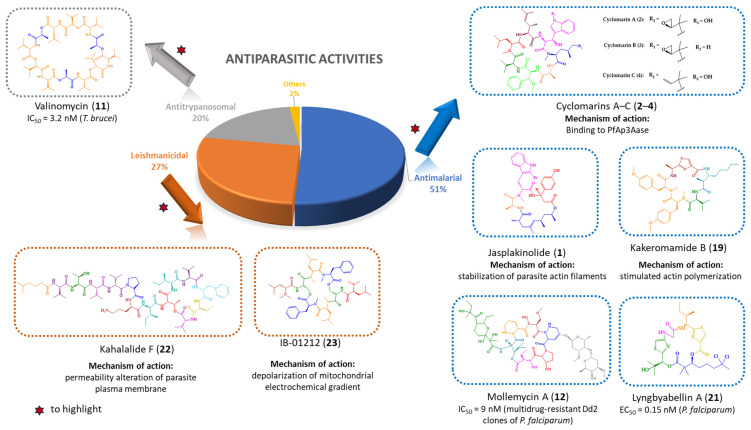
Antiparasitic activities of marine-derived cyclic peptides and their percentages, highlighting the most promising for each activity.

As shown in Figure 11, the most described antiparasitic activity associated with marine-derived cyclic peptides is antimalarial activity (51%) followed by antileishmanial (27%) and antitrypanosomal (20%) activities. It was found that some cyclic peptides were demonstrated to be active against various parasites, such as jasplakinolide (**1**), venturamides A–B (**5**–**6**), symplocamide A (**7**), mollamide B (**8**), valinomycin (**11**), and dudawalamides A–D (**15**–**18**). 

Regarding the antimalarial activity, several promising cyclic peptides were found, specifically jasplakinolide (**1**), cyclomarins A–C (**2**–**4**), mollemycin A (**12**), kakeromamide B (**19**), and lyngbyabellin A (**21**). For some of them, the mechanisms of action have been investigated, exposing different strategies against the parasite. The diverse mechanisms of action and targets are summarized in Figure 11.

Concerning the antileishmanial activity, it is important to emphasize the cyclic peptides kahalalide F (**22**) and IB-01212 (**23**) which displayed high activity against both promastigote and amastigote stages of *Leishmania*. In addition, their mechanisms of action have been explored, highlighting different targets and strategies against the parasite (Figure 11). 

Valinomycin (**11**) is highlighted, considering its potent activity against *T. brucei*.

In addition to antiparasitic effects, some marine-derived cyclic peptides also showed other biological activities, such as anti-inflammatory and anti-tuberculosis (cyclomarin A (**2**)), antibacterial (mollemycin A (**12**)), antifungal (jasplakinolide (**1**), kahalalide F (**22**)), antitumor (symplocamide A (**7**)), antiswarming (lagunamides A–B (**9**–**10**)), and antiviral (valinomycin (**11**)). Moreover, some peptides that are being widely investigated and have demonstrated success for other bioactivities (some of which are already in clinical trials) were subsequently tested for antiparasitic activity. Therefore, exploration of other bioactivities of marine cyclic peptides may constitute a promising route for inspiring the development of new drug molecules.

## 4. Conclusions

In this review, several promising antiparasitic marine-derived cyclic peptides are described, some of which possess unique structural features and relevant activities, resulting from diverse marine sources. Despite being an inspiring area, the research of marine-derived cyclic peptides in this therapeutic area is still scarce. The data compiled in this study are expected to encourage the broadening and dissemination of information about this class of antiparasitic compounds, in addition to supporting future research to better understand their potential and applicability.

## Figures and Tables

**Table 1:** Marine-derived cyclic peptides with antiparasitic effects (**1**–**25**).

Peptides	Types	Sources	Organisms	Country	Antiparasitic Activities	Refs.
Jasplakinolide (**1**)	Cyclic tridepsipeptide	Sponge	*Jaspis* sp.	Island of Benga, Fiji	active against *P. falciparum*, *T. gondii*, *E. histolytica* and *E. invadens*	[127,128,129,130,131]
Cyclomarins A–C (**2**–**4**)	Cyclic heptapeptides	Bacteria	*Streptomyces* sp.	United States (San Diego, CA)	**2**: active against *P. falciparum* **4**: active against multidrug-resistant *P. falciparum* strains	[132,133,134]
Venturamides A–B (**5**–**6**)	Cyclic hexapeptides	Cyanobacteria	*Oscillatoria* sp.	Panama	active against *P. falciparum*, *T. cruzi*, and *L. donovani*	[135]
Symplocamide A (**7**)	Cyclic lipohexadepsipeptide	Cyanobacteria	*Symploca* sp.	Papua New Guinea	active against *P. falciparum*, *T. cruzi*, *L. donovani*	[136]
Mollamide B (**8**)	Cyclic hexapeptide	Tunicate	*Didemnum molle*	Manado Bay, Indonesia	active against *P*. *falciparum* clones and *L*. *donovani*	[137]
Lagunamides A and B (**9**–**10**)	Cyclic pentadepsipeptides	Cyanobacteria	*Lyngbya majuscula*	Pulau Hantu Besar, Singapore	active against *P*. *falciparum*	[138]
Valinomycin (**11**)	Cyclic dodecadepsipeptide	Bacteria	*Streptomyces* sp.	Croatia	active against *P*. *falciparum*, *T. brucei* and *L. major*	[139,140]
Mollemycin A (**12**)	Cyclic glyco-hexadepsipeptide-polyketide	Bacteria	*Streptomyces* sp.	Australia, South Molle Island, Queensland	active against *P*. *falciparum*	[141]
Companeramides A and B (**13**–**14**)	Cyclic octadepsipeptides	Cyanobacteria	*Leptolyngbya* sp.	Coiba Island, Panama	active against *P*. *falciparum*	[142]
Dudawalamides A–D (**15**–**18**)	Cyclic hexadepsipeptides	Cyanobacteria	*Moorea producens*	Papua New Guinean	**15**–**18**: active against *P*. *falciparum* and *L*. *donovani* **18**: active against *T*. *cruzi*	[143]
Kakeromamide B (**19**)	Cyclic pentapeptide	Cyanobacteria	*Moorea producens*	Northern Lau Islands of Fiji	active against *P. falciparum* blood-stage and against *P. berghei* liver schizonts	[144]
Ulongamide A (**20**)	Cyclic tetradepsipeptide	Cyanobacteria	(a) *Lyngbya* sp. (b) *Moorea producens*	(a) and (b) Northern Lau Islands of Fiji	active against *P. falciparum* blood-stages	[144]
Lyngbyabellin A (**21**)	Cyclic tetradepsipeptide	Cyanobacteria	(a) *Lyngbya majuscula* (b) *Moorea producens*	(a) Northern Lau Islands of Fiji (b) Finger’s Reef, Guam	active against *P. falciparum* blood-stages	[144]
Kahalalide F (**22**)	Cyclic dodecadepsipeptide	Mollusk	*Elysia rufescens*	O’ahu, Hawaii	active against *L*. *donovani* promastigotes, *L*. *pifanoi* promastigotes and amastigotes	[145,146,147]
IB-01212 (**23**)	Cyclic octadepsipeptide	Fungus	*Clonostachys* sp. ESNA-A009	Japan	active against *L*. *donovani* promastigotes and *L*. *pifanoi* amastigotes	[148,149]
Janadolide (**24**)	Cyclic pentapolyketidepsipeptide hybrid	Cyanobacteria	*Okeania* sp.	Janado, Okinawa	active against *T. b. brucei*, *T. b. rhodesiense* and *T. cruzi*	[150,151]
Motobamide (**25**)	Cyclic decapeptide	Cyanobacteria	*Leptolyngbya* sp.	Okinawa Island, Japan	active against bloodstream forms of *T. b. rhodesiense*	[152]

*Entamoeba histolytica* (*E. histolytica*), *Entamoeba invadens* (*E. invadens*), *Leishmania donovani* (*L. donovani*), *Leishmania major* (*L. major*), *Leishmania pifanoi* (*L. pifanoi*), *Plasmodium berghei* (*P. berghei*), *Plasmodium falciparum* (*P. falciparum*), *Toxoplasma gondii* (*T. gondii*), *Trypanosoma brucei* (*T. brucei*), *Trypanosoma brucei brucei* (*T. b. brucei*), *Trypanosoma brucei rhodesiense* (*T. b. rhodesiense*), *Trypanosoma cruzi* (*T. cruzi*).

## Data Availability

Not applicable.
